# A novel technique for assessment of post-extubation airway obstruction can successfully replace the conventional cuff leak test: a pilot study

**DOI:** 10.1186/s12871-022-01576-x

**Published:** 2022-02-02

**Authors:** Kentaro Tokunaga, Tadashi Ejima, Takuro Nakashima, Manami Kuwahara, Noriko Narimatsu, Katsuyuki Sagishima, Teruhiko Mizumoto, Takuro Sakagami, Tatsuo Yamamoto

**Affiliations:** 1 Department of Intensive Care Medicine, Kumamoto University Hospital, 1-1-1 Honjo, Chuo-Ku, Kumamoto, 860-8556 Japan; 2 Department of Respiratory Medicine, Kumamoto University Hospital, 1-1-1 Honjo, Chuo-Ku, Kumamoto, 860-8556 Japan; 3 Department of Anesthesiology, Kumamoto Rosai Hospital, 1670 Takehara-machi, Yatsushiro-shi, Kumamoto, 866-8533 Japan; 4 Department of Nephrology, Graduate School of Medical Sciences, Kumamoto University, 1-1-1 Honjo, Chuo-Ku, Kumamoto, 860-8556 Japan

**Keywords:** Intensive care, Cuff leak test, Post-extubation edema, Post-extubation stridor, Patient-ventilator asynchrony

## Abstract

**Background:**

Post-extubation airway obstruction is an important complication of tracheal intubation. The cuff leak test is traditionally used to estimate the risk of this complication. However, the cuff leak test parameters are not constant and may depend on the respiratory system and ventilator settings. Furthermore, deflating the cuff also be a risk factor for patient-ventilator asynchrony and ventilator-associated pneumonia.

Instead of using the cuff leak test, we measured the pressure of the leak to the upper airway through the gap between the tube and glottis with a constant low flow from the lumen above the cuff without deflating the cuff and called it "pressure above the cuff."

The purpose of this study was to investigate whether pressure above the cuff can be used as an alternative to the cuff leak volume.

**Methods:**

This prospective observational study was conducted at Kumamoto University Hospital after obtaining approval from the institutional review board. The pressure above the cuff was measured using an endotracheal tube with an evacuation lumen above the cuff and an automated cuff pressure modulation device. We pumped 0.16 L per minute of air and measured the steady-state pressure using an automated cuff pressure modulation device. Then, the cuff leak test was performed, and the cuff leak volume was recorded. The cuff leak volume was defined as the difference between the expiratory tidal volume with the cuff inflated and deflated. The relationship between the pressure above the cuff and cuff leak volume was evaluated. The patient-ventilator asynchrony during each measurement was also examined.

**Results:**

The pressure above the cuff was measured, and the cuff leak volume was assessed 27 times. The pressure above the cuff was significantly correlated with the cuff leak volume (*r* = -0.76, *p* < 0.001). Patient-ventilator asynchrony was detected in 37% of measurements during the cuff leak test, but not during the pressure above the cuff test.

**Conclusions:**

This study suggests that pressure above the cuff measurement may be a less complicated alternative to the conventional cuff leak test for evaluation of the risk of post-extubation airway obstruction.

**Trial registration:**

University Hospital Medical Information Network Clinical Trials Registry (UMIN000039987; March 30, 2020). https://upload.umin.ac.jp/cgi-open-bin/ctr_e/ctr_view.cgi?recptno=R000044604

## Background

Post-extubation laryngeal edema and post-extubation airway obstruction are some of the complications following extubation that can cause reintubation. The presence of laryngeal edema increases the duration of intubation [1]. Post-extubation stridor is a clinical sign of laryngeal edema. The reported prevalence of post-extubation airway obstruction is 4–37% [2].

One of the common methods for predicting post-extubation airway obstruction is the cuff leak test (CLT) [1–4]. This test is used to evaluate the leak around the endotracheal tube when the cuff is deflated. For this purpose, qualitative and quantitative methods are used. A cuff leak absolute volume less than 110 mL is one of the thresholds for CLT [3]. However, the most accurate way to validate CLT is unknown, and no evaluation criteria have been established [4]. Additionally, CLT can induce coughing [3], and this may result in errors in exhaled tidal volume measurements [5, 6]. Furthermore, respiratory system compliance may affect CLT results [7]. Although mechanical ventilation settings, such as positive end expiratory pressure (PEEP) and tidal volume, can affect the cuff leak volume, ventilator settings differed among previous studies [3, 5, 8–11].

In some studies, CLT has been considered a noninvasive and easy method for predicting post-extubation airway obstruction [5, 12–14]. However, circuit leakage causes patient-ventilator asynchrony [15–18]; therefore, CLT can cause patient-ventilator asynchrony. Several studies that referred to ventilator settings during CLT used volume assist-control [5, 12–14, 19]. Volume assist-control is more likely to cause asynchrony than other ventilator modes, such as pressure assist-control and pressure support [20]. CLT also increases the risk of subglottic secretion leakage into the lower airway and ventilator-associated pneumonia (VAP) during cuff deflation [21].

To address these concerns, a different evaluation method than the conventional CLT is needed. For this purpose, we developed a new technique, the pressure above the cuff test. In this test, the pressure is measured without deflating the cuff at the beginning of air leakage from the lumen above the cuff into the larynx through the gap between the tube and the glottis. Pressure is measured in the suction evacuation line above the endotracheal tube cuff. We hypothesized that if the pressure is low, we can infer that there is no obstruction in the upper airway, while high pressure suggests airway obstruction or narrowing. We sought to determine whether the new method is related to conventional CLT.

## Methods

### Study design and ethics

This single-center observational pilot study enrolled 25 patients admitted to the intensive care unit (ICU) of Kumamoto University Hospital between April 2020 and July 2020. The sample size was determined based on the feasibility of the study. Before data collection, the study protocol was reviewed and approved by the Kumamoto University Research Ethics Committee (approval number: 2500) and the study was registered in the University Hospital Medical Information Network Clinical Trial Registry System (UMIN-CTR, ID: 000,039,987). No patients or staff members with coronavirus disease 2019 were present in the hospital during this period.

Patients ventilated in the ICU with an endotracheal tube with an evacuation lumen that was open above the cuff for suction of secretions accumulated onto the cuff (Portex Blue Line SACETT®, Smiths Medical ASD Inc.; Weston, MA, USA) were included. The exclusion criteria included: inability to obtain informed consent from the patients or their relatives, mental instability such as delirium, use of a mechanical circulatory assist device, and declination of enrolling the patient by the clinician-in-charge.

Intubation was performed in the operating room or the ICU, and the type and size of the endotracheal tube was determined by the intubating clinician. Informed consent was obtained from the patients who met the inclusion criteria or their relatives. All recruited patients were intubated via the oral route. The cuff pressure was maintained at 30 cm H_2_O using an aneroid manometer.

### Above-cuff leak test

**Fig. 1:**
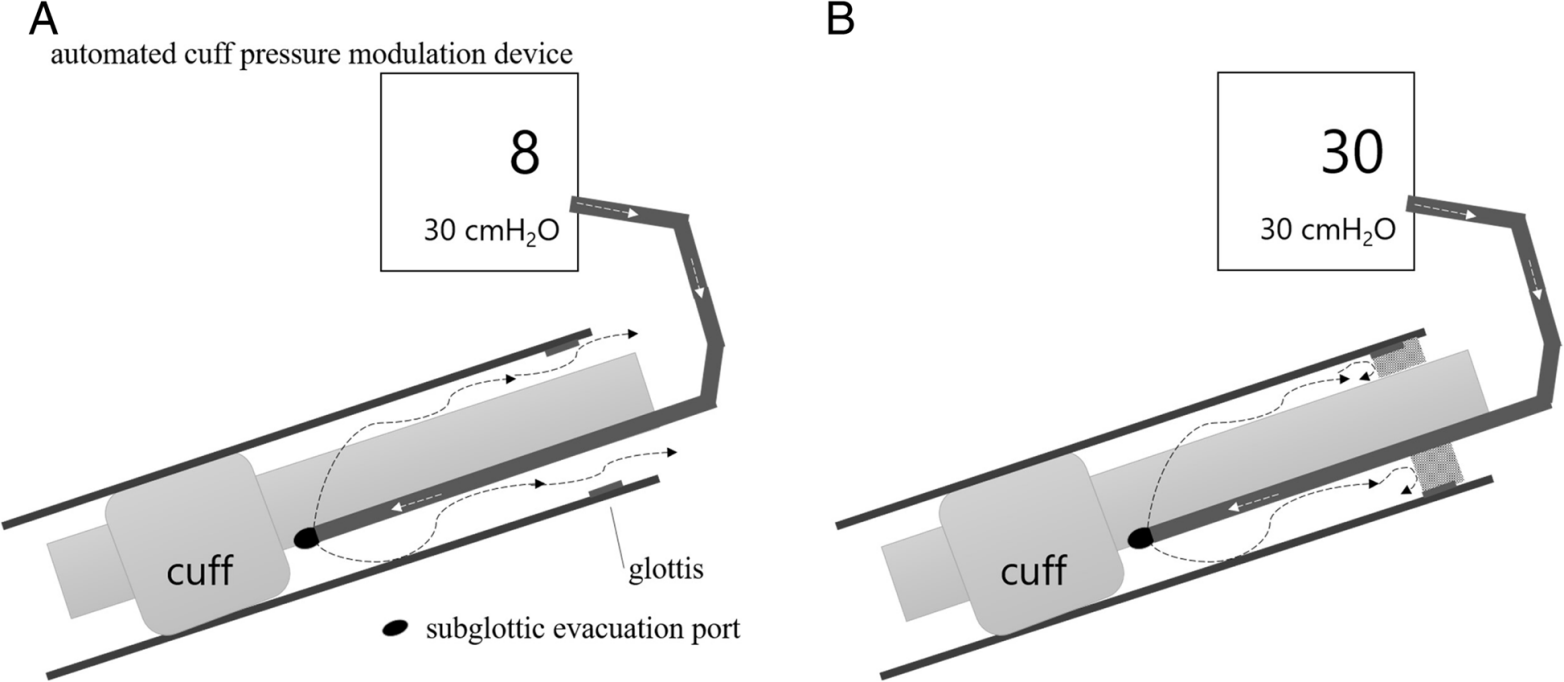
Schema of the above-cuff leak test. The smaller number at the bottom of the screen of the automated cuff pressure modulation device is the set pressure, and the larger number is the value of the measured pressure. Air (⇢) is delivered from the automated cuff pressure modulation device through the evacuation line to the subglottic evacuation port. Then, the air flow leaks to the upper airway through the gap between the tube and the glottis with an intact upper airway (**A**). At that time, the measured pressure became smaller than the set pressure. If there was upper airway obstruction, such as laryngeal edema, the air flow did not leak into the upper airway and the set and measured pressures were equal (**B**)

The ventilator settings were left unchanged at the time of the above-cuff leak test. In this test, air was delivered to the upper respiratory tract without deflating the cuff (Fig. 1). We measured the plateau pressure of the upper airway through the gap between the tube and the glottis with a constant low flow (0.16 L per minute) from the lumen above the cuff. An automated cuff pressure modulation device (Mallinckrodt Pressure Control®; VBM Medizintechnik GmbH, Sulz am Neckar, Germany) was used to generate the flow and measure the pressure.

Before the above-cuff leak test measurement, oral, endotracheal, and subglottic secretions were suctioned. In addition, air was infused into the subglottic evacuation line with a syringe to ensure that it was not obstructed. The cuff pressure modulation device was connected to the subglottic evacuation line of the cuff. The measured value of pressure, which was digitally displayed by the cuff pressure modulation device, was recorded as “the pressure above the cuff.” The cuff pressure was 30 cm H_2_O; this pressure was equal to the pressure of the cuff pressure modulation device to avoid exceeding the pressure in the cuff. Therefore, the maximum value of "the pressure above the cuff" was 30 cm H_2_O. The mechanical ventilator settings did not change during the procedure.

### Cuff leak test

The CLT was conducted after the above-cuff leak test. The mechanical ventilator was changed to the volume assist-control mode. The Servo I® ventilator (Maquet, Solna, Sweden) was used in all patients. The respiratory rate and tidal volume were adapted for patient comfort by referring to the state before the setting change. The PEEP level was the same as that before the test. Initially, we measured three subsequent breath cycles and averaged the three expiratory tidal volumes with the cuff inflated. At this time, the peak airway pressure was recorded. The cuff was deflated. After six subsequent breath cycles, the lowest three expiratory tidal volumes were then averaged. This procedure was performed according to the protocol proposed by Miller and Cole [3]. The difference between the expiratory tidal volumes with the cuff inflated and deflated was defined as the cuff leak volume. The actual tidal volume at expiration was measured before deflation and with deflation of the endotracheal tube cuff [11, 22, 23].

### Auscultation cuff leak test [11]

During cuff deflation, we assessed the leaking sound and classified it. No leak was defined as no sound of leak heard using a stethoscope; mild leak was defined as the presence of a leak sound heard using a stethoscope; large leak was defined as the presence of sound of leak heard without using a stethoscope.

### Patient-ventilator asynchrony

**Fig. 2:**
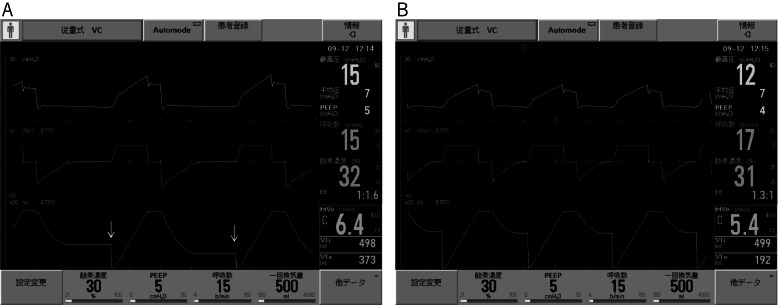
Graphical examples of patient-ventilator asynchrony. Airway pressure (top), flow (middle), and volume (bottom) waveforms over time during volume-assisted control ventilation. The respiratory rate and tidal volume were set to 15 breaths/min and 500 mL, respectively. **A** With the cuff deflating, the expiratory tidal volume (VTe) was lower than the inspiratory tidal volume (VTi). The expiratory volume waveforms fall and then plateau without reaching zero (arrows); **B** All cycles occurring at a respiratory rate of 17 breaths/min, which exceeds the set frequency, were auto-triggered due to circuit leakage

The investigators determined patient-ventilator asynchrony by observing the graphical screen of the ventilator during the CLT (Fig. 2) [16–18]. Asynchrony was determined by ICU staff familiar with ventilator management.

### Other measurements

The following patient parameters were determined: age, sex, height, weight, body mass index, characteristics of intubation (emergency or planned for surgery), endotracheal tube diameter, and presence of post-extubation stridor.

### Analysis

For continuous variables, mean ± standard deviation or median and 25% and 75% percentile values were calculated, while categorical variables were expressed as number (%). Correlations between the pressure above the cuff and cuff leak volume were evaluated using Spearman’s rank correlation coefficient. Auscultation CLT and pressure above the cuff results were compared using the Mann–Whitney U test. Statistical analyses were performed using EZR (Saitama Medical Center, Jichi Medical University, Saitama, Japan), which is a graphical user interface for R (The R Foundation for Statistical Computing, Vienna, Austria). More precisely, it is a modified version of R commander that includes statistical functions that are frequently used in biostatistics. Statistical significance was set at *P* < 0.05.

### Monitoring and auditing

Monitoring was performed every five cases to assess adherence to the trial protocol, accuracy of completed case report forms, and the electronic dataset. Monitoring was performed at the Center for Clinical Research at Kumamoto University Hospital. The principal investigator agreed to allow inspectors from regulatory agencies to review records and assist the inspectors in their duties, if requested.

## Results

### Baseline characteristics

The tests were performed in all the 25 included patients. The tests were repeated in two patients: one patient showed no leak during CLT the first time, but was tested again due to an early extubation schedule, while another patient showed a leak during CLT the first time, but was tested again later because extubation was postponed. Table 1 summarizes the clinical characteristics of the included patients.

**Table 1:** Patient's characterisitics

Characterisitics	
Gender M/F	17/8
Age (years)	67 (59–73)
Body mass index (kg/m^2^)	24.0 (22.8–26.1)
Place of intubation	
Operation room	23 (92)
Intensive Care Unit	2 (8)
Postoperative cardiovascular surgery	18 (72)
Tube diameter (mm)	8.00 (7.00–8.00)
7	8 (32)
7.5	4 (16)
8	14 (56)
8.5	1 (4)
PEEP (cmH_2_O)	5.0 (5.0–6.5)
5	19 (76)
6	1 (4)
7	1 (4)
8	4 (16)
9	0 (0)
10	2 (8%)
Ventilator settings during the above-cuff leak test	
Pressure-controlled SIMV with PS	21 (84)
CPAP with PS	6 (24)
PC or PS (cmH2O)	13 (10–15)
Respiratory parameters during CLT	
Peak inspiratory pressure (cmH2O)	23.0 (20.0–25.5)
Tidal volume (ml)	450(410–500)
TV/PBW	7.9(7.1–8.8)
RASS	-4.1 (± 1.2)

Data are presented as median (interquartile) or median (± SD) for continuous variables, as appropriate, and as number (%) for categorical variables

*PEEP* positive end-expiratory pressure, *SIMV* synchronized intermittent mandatory ventilation, *PS* pressure support, *PC* pressure control, *CPAP* continuous airway positive pressure, *TV/PBW* tidal volume/predicted body weight, *RASS* Richmond Agitation Sedation Scale, *CLT* cuff leak test

### Cuff leak test and pressure above the cuff

**Fig. 3:**
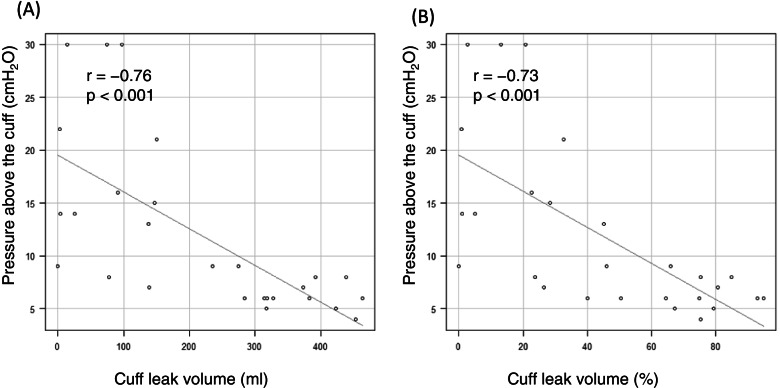
Correlations between pressure above the cuff and cuff leak volume

The PEEP was the same as that before the test. The median PEEP was 5 cmH2O, and an additional PEEP of 8 cmH2O was used in 24% of the patients (Table 1). No post-extubation stridor occurred in this study (Table 2). The median pressure above the cuff was 12 cmH_2_0. The minimum pressure above the cuff in this study was 4 cmH_2_0. The pressure above the cuff correlated significantly with the cuff leak volume [absolute value of cuff leak volume, *r* = -0.76, *p* < 0.001 (Fig. 3A); relative value of cuff leak volume, *r* = -0.73, *p* < 0.001 (Fig. 3B)].

**Table 2:** Comparison between the above-cuff leak test and cuff leak test

n (times)	27
Stridor	0
Pressure above the cuff (cmH2O)	8 (6.0–14.5)
Tidal volume (mL)	
pre-CLT	500.0 (420.5–523.5)
post-CLT	277.0 (135.5–372.3)
Cuff leak volume (%)	45.1 (21.7–75.0)
Cuff leak volume (mL)	235 (84.5–350.5)
Auscultation CLT	
large	21 (77.8)
mild	3 (11.1)
no	3 (11.1)

Data are presented as median (interquartile) or median (± SD) for continuous variables, as appropriate, and as number (%) for categorical variables

Cuff leak volume (%) = 100 × (tidal volume with cuff inflated − tidal volume with cuff deflated) / tidal volume with cuff inflated

*CLT* cuff leak test

**Fig. 4:**
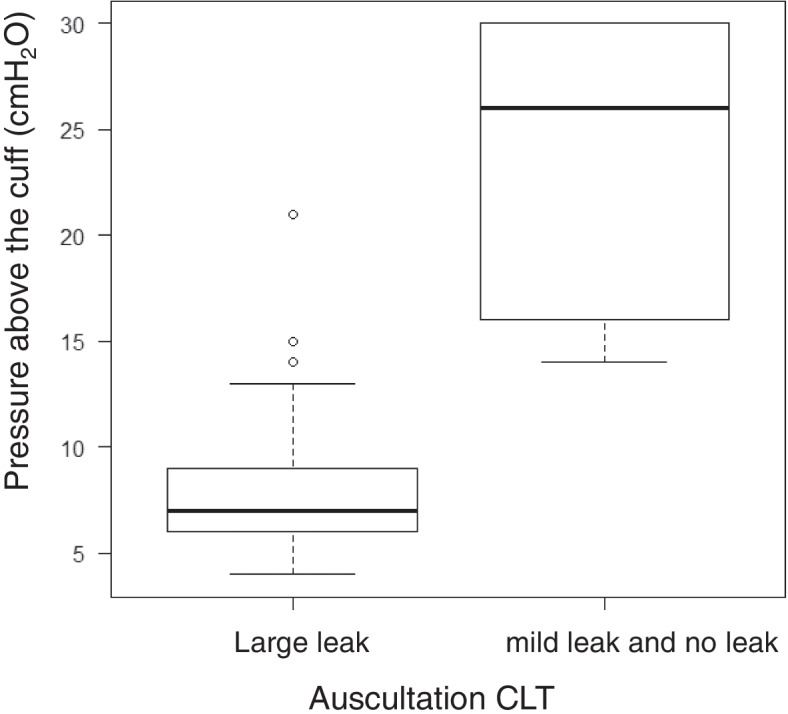
Relationship between auscultation cuff leak test and pressure above the cuff test *p* < 0.01

The pressure above the cuff correlated with the auscultation CLT. A large leak heard on auscultation CLT was associated with a lower pressure above the cuff (*p* < 0.001; Fig. 4).

Patient-ventilator asynchrony was detected in 37% of the patients during conventional CLT. However, asynchronous events did not occur during the above-cuff leak test. All asynchrony episodes were caused by auto-triggering.

We performed a subgroup analysis of 17 episodes, excluding 10 episodes of patient-ventilator asynchrony. The pressure above the cuff correlated significantly with the cuff leak volume [absolute value of cuff leak volume, *r* = -0.78, *p* < 0.001; relative value of cuff leak volume, *r* = -0.77, *p* < 0.001]. A large leak heard on auscultation CLT was associated with a lower pressure above the cuff (*p* < 0.001).

## Discussion

This study compared the potential of post-extubation stridor detection between the pressure above the cuff test and the CLT. Results suggest that low pressure above the cuff value correlates with a low cuff leak volume. Because no post-extubation stridor occurred in this study, this study only examined the relationship between the pressure above the cuff and the cuff leak volume, and not the relationship between the pressure above the cuff and post-extubation airway obstruction. Although stridor did not occur in the present study, the cuff leak volume is used as one of the assessment variables for post-extubation airway obstruction. The pressure above the cuff might be a potential less invasive alternative method.

Prinianakis et al. showed that respiratory system dynamics and inspiratory flow affect inspiratory leakage and may contribute to the poor performance of the CLT; however, these have not been taken into account in previous studies [7]. In our study, the above-cuff leak test did not affect respiratory system dynamics and inspiratory flow, because the measurements were performed at a site unrelated to ventilation and the cuff was not deflated. In addition, theoretically, the CLT also depends on other factors, such as exhalation effort, expiratory flow rate, expiratory time, and air trapping. However, these factors did not affect the above-cuff leak test results. CLT was performed using the volume assist-control mode in this study. This is the main mode of ventilation that was used in past CLT studies [2, 5, 12–14, 19]. Antonaglia et al. performed CLT with CPAP; the cuff leak volume was smaller than that in other studies [24]. The cuff leak volume may change if the ventilation volume or flow is different or if there is air intake from around the cuff due to respiratory effort. In other words, if the mode of ventilation is different, the correlation may differ from that in this study.

Several studies have reported that laryngeal air column width measured by laryngeal ultrasonography is a predictive indicator of airway obstruction after extubation [5, 19, 23, 25]. Another study examined the correlation between laryngeal air column width and the cuff leak volume and found no correlation (*r* = 0.20; P = 0.051) [5]. However, since ultrasonography results depend on the skill of the examiner, the above-cuff leak test can be more accessible to predict post-extubation airway obstruction because it is technically simpler than laryngeal ultrasonography.

The tube diameter will affect the cuff leak volume and the pressure above the cuff respectively.

Cuff deflation can cause circuit leakage and auto-triggering. Auto-triggering is a type of patient-ventilator asynchrony [15]. Deflating the cuff should allow auto-triggering to occur, but its effects have not been evaluated in previous CLT studies. Auto-triggering may underestimate or overestimate the expiratory flow, because inspiration may be triggered without finishing the exhalation or the next inhalation may occur after a premature exhalation. Furthermore, cuff deflation may cause a drop in the secretions. Secretions leaking into the lungs across the cuff of the endotracheal tube is considered a pathogenic mechanism of VAP [26]. Since the above-cuff leak test does not require cuff deflation, no auto-triggering occurs and VAP is unlikely to occur. Moreover, the lack of the need of cuff deflation means that the test can be easily repeated.

The American Thoracic Society/American College of Chest Physicians guidelines state that failing to perform the CLT might lead to a delay in extubation [27]. Because measurement of the pressure above the cuff is simpler than the CLT and can be repeated as needed, the above-cuff leak test may prevent extubation delays.

This study had some limitations. First, the study investigators were not blinded to the clinical information when interpreting the test results. Therefore, they may have introduced recall bias. Second, there are other automated cuff pressure modulation devices, in addition to the one used in this study. Further, there is more than one type of product and other similar products besides the one used in this study. Therefore, the obtained results may not be reproduced using other devices. Third, the effect of stenosis at the cuff contact on the pressure above the cuff measurement was not assessed. However, since cuff pressure modulation device low-pressure cuffs have been used, the incidence of postintubation tracheal stenosis at the site of the cuff is rare [28]. Fourth, the tube diameter will affect the pressure above the cuff. In the present study, the size of the endotracheal tube ranged from 7–8.5 mm. The same patient could have had different pressures above the cuff if the tube size was different. It is nearly impossible to change the size of the endotracheal tube and recheck the pressure above the cuff in the same patient. There is a potential limitation on the prediction capacity of both tests by the influence of tube size on the pressure above the cuff and cuff leak volume. Fifth, the pressure level of the cuff pressure modulation device might not have reached the true minimum pressure level during pressure above the cuff measurement because of resistance in the extension tube. Finally, although different researchers conducted the tests in this study, the inter-observer reproducibility was not assessed. However, we attempted to overcome this bias by standardizing the technique described in the Methods section.

## Conclusions

This study indicated that pressure above the cuff measurement may be a less complicated alternative to conventional CLT. Further large-scale studies are needed to determine the threshold for the above-cuff leak test and to investigate the association between post-extubation airway obstruction and the pressure above the cuff.

## Data Availability

The datasets used and/or analyzed during the current study are available from the corresponding author upon reasonable request.

## Abbreviations

CLT: Cuff leak test
ICU: Intense care unit
PEEP: Positive end-expiratory pressure
VAP: Ventilator-associated pneumonia
